# Ablation Strategies for Persistent Atrial Fibrillation: Beyond the Pulmonary Veins

**DOI:** 10.3390/jcm13175031

**Published:** 2024-08-25

**Authors:** Omar Baqal, Areez Shafqat, Narathorn Kulthamrongsri, Neysa Sanghavi, Shruti K. Iyengar, Hema S. Vemulapalli, Hicham Z. El Masry

**Affiliations:** 1 Department of Cardiovascular Medicine, Mayo Clinic, Phoenix, AZ 85054, USA; thames.kulthamrongsri@gmail.com (N.K.); iyengar.shruti@mayo.edu (S.K.I.); vemulapalli.hema@mayo.edu (H.S.V.); 2 College of Medicine, Alfaisal University, Riyadh 11533, Saudi Arabia; ashafqat@alfaisal.edu; 3 St. George’s University School of Medicine, West Indies P.O. Box 7, Grenada; neysa.sanghavi@gmail.com

**Keywords:** persistent atrial fibrillation, catheter ablation, pulmonary vein isolation, posterior wall isolation, non-pulmonary vein triggers, substrate modification

## Abstract

Despite advances in ablative therapies, outcomes remain less favorable for persistent atrial fibrillation often due to presence of non-pulmonary vein triggers and abnormal atrial substrates. This review highlights advances in ablation technologies and notable scientific literature on clinical outcomes associated with pursuing adjunctive ablation targets and substrate modification during persistent atrial fibrillation ablation, while also highlighting notable future directions.

## 1. Introduction

Pulmonary vein isolation (PVI) during catheter ablation is a well-established treatment strategy for paroxysmal atrial fibrillation (AF) [1]. However, persistent AF (PeAF) still exhibits less favorable outcomes despite advances in ablative therapies; large-scale randomized trials demonstrate that only 50–60% of PeAF patients maintain sinus rhythm following a single ablation procedure [2,3,4]. Success rates for long-standing PeAF (>12 months) are even lower [4,5]. This variability in treatment response is explained by the fact that paroxysmal AF is mainly driven by pulmonary vein triggers. In contrast, the efficacy of PVI for PeAF is often hindered by the presence of non-pulmonary vein triggers (NPVTs) and abnormal atrial substrates. The presence of NPVTs, including sites like the left atrial (LA) posterior wall (PW), LA appendage (LAA), ligament of Marshall, coronary sinus, superior vena cava, crista terminalis, and even premature atrial complexes (PAC) when using a broader definition, is pivotal in both initiating and advancing disease into PeAF [6]. Thus, PVI alone may not achieve optimal outcomes in PeAF [5].

Catheter ablation techniques are employed to identify and eliminate these NPVTs, attenuating the propensity for AF recurrence. Techniques employed in substrate ablation include the creation of linear ablation lesions, ablation of complex fractionated atrial electrograms (CFAEs), or focal impulse and rotor modulation (FIRM)-guided ablation. However, the clinical impact of pursuing adjunctive ablation of targets in PeAF remains controversial (Table 1 and Table 2). For instance, the STAR AF II trial, which compared PVI, PVI + CFAEs, and PVI + linear ablation, showed no reduction in AF recurrence with adjuvant substrate modification among patients with PeAF [2]. Although empiric ablation of additional targets may benefit select patients, the overall success is suboptimal, and there is a need to formulate a tailored approach to ablation target selection.

Patients with AF recurrence post-ablation are considered for repeat ablation, although guidelines and evidence on the optimal approach to re-ablation and target selection are far less informed than with index ablation [7]. This is especially true for PeAF, where data on the effectiveness of re-ablation is limited to observational studies. Barakat et al. noted that while re-ablation significantly reduced arrhythmia recurrence in PeAF patients compared to medical therapy, recurrence with re-ablation was still quite high, at around 60% [8]. Much of the guidance on re-ablation pertaining to NPVTs and substrate modification is extrapolated from studies on index ablation, which must be interpreted with caution. The timing of re-ablation and the optimization of risk factors for AF recurrence are important aspects of procedural planning that may also influence ablation outcomes.

The updated AF management guidelines outline the role of pharmacological cardioversion and maintenance of sinus rhythm in patients who decline or are not candidates for ablation or prefer antiarrhythmic therapy [9]. In a systematic review of six RCTs (*n* = 1212), catheter ablation was found to be associated with lower recurrent atrial arrhythmia, lower hospitalizations, and similar serious adverse event rates when compared to anti-arrhythmic drugs (AADs) [10]. Nevertheless, AADs play an important role in keeping patients out of AF and limiting the likelihood of left atrial (LA) remodeling, which in turn drives AF persistence. Investigations into the role of autonomic dysfunction and neurohormonal disorders in heart failure have led to the conceptualization of the role of neurohormonal blockade in facilitating reverse cardiac remodeling that may be beneficial in reducing the risk of AF onset and recurrence. Nuzzi et al. studied the LA volume index (LAVI) among 560 patients with dilated cardiomyopathy, noting a lower risk of death, heart transplantation, and heart failure hospitalization among patients with high reductions in LAVI over follow-up [11]. Maintenance of sinus rhythm, whether through ablation, pharmacotherapy, or both, can have significant short- and long-term prognostic implications for AF patients. 

This review explores the contemporary literature on evolving ablative treatment strategies for PeAF, including novel ablation technologies and clinical outcomes associated with pursuing adjunctive ablation targets beyond the PVs. 

## 2. Ablation Strategies 

### 2.1. Radiofrequency Ablation and Cryoablation 

Radiofrequency ablation (RFA) involves applying high-frequency electrical currents to generate localized heat, forming controlled lesions within the cardiac tissue. This targeted energy deposition induces scar formation, thereby interrupting aberrant electrical triggers responsible for perpetuating AF. The technique is favored for its efficacy and relatively low incidence of procedural complications [12].

Conversely, during cryoablation, tissue freezing is induced, and subsequent cellular apoptosis occurs along the catheter–tissue interface. Cryoablation represents an alternative approach to AF ablation and has been used to target both the PVs as well as the PW for isolation with equivalent procedural outcomes [12]. 

In the STOP persistent AF trial, just over half (55%) of drug-refractory PeAF patients who underwent a cryoballoon PVI remained free of AF/AT for 12 months, with a low rate of adverse events [13]. Straube et al. noted a much higher freedom from AF/AT at 12 months (82%) among PeAF patients undergoing cryoballoon PVI (*n* = 1140), with a 1.5% major complication rate [14]. An international multicenter registry (*n* = 609) noted a freedom from AF/AT of 64% and 57% for PeAF and long-standing PeAF at 24 months of follow-up, respectively [15]. When comparing cryoablation to RFA, a study on PeAF found no significant difference in arrhythmia relapse between RFA and cryoablation during short- (≤3 months) and mid-term (3 to 12 months) follow-ups. Still, cryoablation had a shorter procedure duration [16]. Another study of 127 patients with PeAF indicated similar effectiveness for both methods over 36 months, though RFA with pressure control catheters had a small but statistically insignificant long-term advantage [17]. These findings support the effectiveness of both methods, with RFA offering more durable results and CBA providing procedural efficiency and safety. In agreement with these findings, a meta-analysis reported comparable safety and efficacy profiles of CBA and RFA, though the former had shorter procedure times (mean reduction 43.77 min, 95% CI 66.45—21.09, *I*^2^) but higher rates of phrenic nerve paralysis (RR, 4.13; 95% CI, 1.49—11.46) [18]. 

### 2.2. Pulsed-Field Ablation

Pulsed-field ablation (PFA) is a nonthermal technique that has revolutionized the approach to AF ablation. Unlike conventional thermal methods, PFA employs high-frequency pulsed electrical fields to induce irreversible electroporation (IRE; i.e., impairing cell membrane integrity and causing cell death) in tissues, bypassing the risks associated with heat-based procedures [19]. 

The distinct advantage of PFA lies in its ability to swiftly create nonthermal cardiac lesions with minimal complications. By selectively targeting cardiomyocytes while sparing surrounding tissues, PFA ensures lesion formation, reducing the risk of adverse events, such as atrial–esophageal fistula formation, phrenic nerve injury, and PV stenosis [20]. Despite initial challenges in targeting tissues accurately and understanding cellular mechanisms, PFA has demonstrated promise in preferentially affecting cardiomyocytes, showcasing its potential for widespread clinical application [19]. 

Various studies have demonstrated the safety and efficacy of PFA. The FARA-Freedom Study reported that 66.6% of patients with paroxysmal AF remained arrhythmia-free 12 months after a single PFA procedure using a pentaspline catheter, with a low incidence of adverse events (1.1%), including one cardiac tamponade, one TIA, and four cases of transient phrenic nerve palsy [21]. Procedural and fluoroscopy times were 71.9 ± 17.6 min and 11.5 ± 7.4 min, respectively [11]. A multi-center MANIFEST-PF Registry study reported a 78.1% success rate in maintaining sinus rhythm 12 months post-procedure in 1568 PeAF patients, with a 1.9% complication rate (18 cardiac tamponade and 6 stroke) [22]. A PULSED AF pivotal trial involving 300 patients with either paroxysmal or PeAF reported an efficacy of 55.1% of PFA in PeAF patients, which was comparable to that of traditional ablation techniques but with significantly fewer complications (0.7%, 1 stroke, 1 cardiac tamponade/perforation) [23]. Focusing on left atrial posterior wall isolation (LAPWI), a study using PFA demonstrated its feasibility and safety and high rates of acute isolation with favorable side effect profile, particularly in avoiding thermal injury to the esophagus [24]. These findings suggest that PFA, with its nonthermal energy delivery, represents a significant advancement in catheter ablation for AF.

However, like any new technology, our understanding of potential limitations and challenges associated with PFA continues to evolve. Based on the current state of investigation, durability, especially long-term, has not been proven to be superior to conventional thermal ablation [25,26,27,28,29]. With regard to lesion quality, it is emerging that close electrode–tissue proximity is important in achieving effective lesions [30,31,32]. Additionally, PFA appears to have minimal effect on the cardiac ganglionated plexi, unlike thermal ablation, thus representing a limited role in the modulation of the cardiac autonomic nervous system [33,34,35]. A recent meta-analysis by Aldaas et el. comparing PFA with thermal ablation demonstrated that, despite the theoretically better safety profile, PFA was associated with increased fluoroscopy time and no significant difference in periprocedural complications, likely due to lack of operator experience with a newer catheter [36]. This hypothesis is further supported by the fact that the MANIFEST-17K study revealed that the incidence of major complications like cardiac tamponade had > 50% reductions at centers that had previously participated in the MANIFEST PF trial [37], indicating that such complications may be rare with greater operator familiarity with the technology. 

With increasing adoption and growing outcome data, potential complications are coming to the fore. For instance, acute kidney injury secondary to acute hemolysis after PFA procedure has been reported [37]. Another potential complication that has been associated with PFA is coronary vasospasm, particularly in the areas adjacent to coronary arteries, which can be attenuated by prophylactic use of nitroglycerin [38]. Larger studies on the neurocognitive effects of micro-bubbles caused by PFA are needed [39,40]. Due to sub-optimal integration between current catheter technologies and mapping systems, tracking errors may be experienced after PFA delivery [41]. 

In conclusion, while PFA epitomizes a significant advancement in AF catheter ablation, further research is needed to assess its safety, durability, and efficacy in the context of PeAF ablation. 

### 2.3. Hybrid Ablation

In PeAF, the myocardium undergoes anatomical and electrophysiological changes due to long-standing AF, which often leads to the development of NPVTs [42]. Arguably the most influential trigger is the LAPW, which is an anatomically challenging area to conduct endocardial ablation and substrate modification due to the risk of cardiac perforation, tamponade, or potential collateral damage to adjacent structures like the esophagus. This is due to its thin nature, especially in patients with long-standing AF with a dilated and remodeled left atrium [43]. It was thought that esophageal temperature sensing during ablation could reduce the risk of damage to the esophagus, but the OPERA trial reported similar incidences of endoscopically diagnosed esophageal lesions regardless of using temperature probes [44]. It was also noted that the LAPW reconnection rate was as high as 40% in patients undergoing a repeat ablation after index endocardial PWI, with the roof of LA being the most common site for reconnection [45]. Currently, there is a scarcity of compelling evidence to support catheter-based adjuvant PWI to PVI as being more effective than PVI alone for preventing arrhythmia recurrence [46,47].

**Figure 1:**
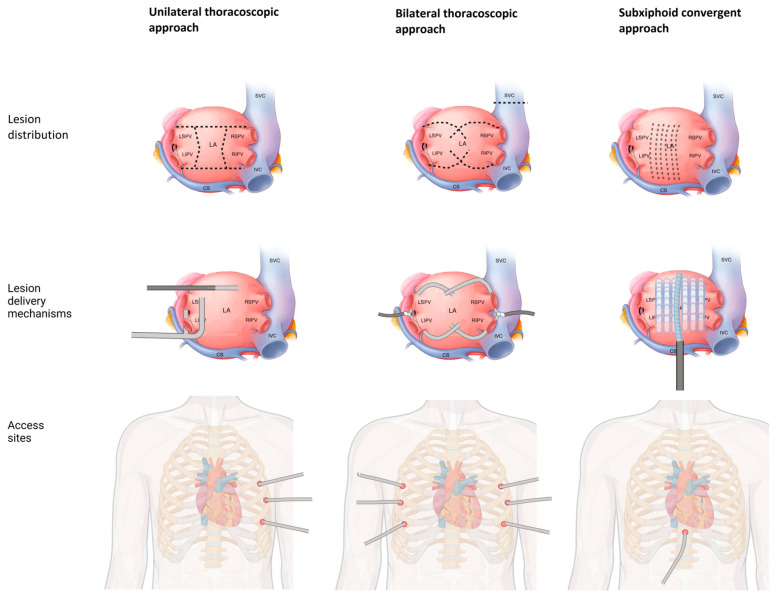
Approaches and procedural characteristics of hybrid ablation.

These limitations led to the development of a hybrid ablation strategy consisting of a minimally invasive epicardial ablation by a cardiothoracic surgeon and an endocardial ablation by an EP specialist to effectively isolate the LAPW transmurally (Figure 1). The 2023 AHA guidelines on the management of AF assign hybrid ablation a class 2b (LOE of B-R) recommendation in patients with symptomatic PeAF that is refractory to AADs [9]. In patients undergoing cardiac surgery, concomitant surgical ablation can be beneficial in reducing recurrence risk (2a, B-R) [9]. The American Association for Thoracic Surgery (AATS) guideline assigns a Class IIa recommendation to concomitant surgical ablation for AF, citing improvement in health-related quality of life (HRQL), AF-related symptoms, and short-term operative survival, with no effect on perioperative morbidity, risk of perioperative stroke/TIA, and long-term survival [48]. Concomitant ablation during cardiac surgery has been studied in various settings, including minimally invasive valve surgery, mitral and non-mitral valve surgery, and CABG, and is associated with overall improved short- and long-term outcomes [49]. The PRAGUE-12 trial (*n* = 206) noted a lower incidence of stroke and AF recurrence with concomitant surgical ablation during cardiac surgery compared to cardiac surgery alone [50]. Gu et al. retrospectively studied 1028 patients undergoing surgical ablation during cardiac surgery, noting improvements in the NYHA class, LV ejection fraction, LA and right atrial diameters, and LV end-diastolic diameter [51]. 

In general, hybrid ablation can be a staged procedure conducted sequentially or a single procedure done in a single session. The staged approach has the advantages of being logistically easier and of identifying lesion gaps after they heal from the epicardial part. In contrast, a single procedure requires specialized hybrid theatres [52].

There are two strategies of hybrid ablation based on surgical access to the pericardium, namely thoracoscopic and subxiphoid/transdiaphragmatic access (hybrid convergent procedure). 

#### 2.3.1. Thoracoscopic Ablation 

Bilateral thoracoscopic ablation can be performed using a single clamping device with an irrigated bipolar RF catheter, where clamps are placed in the oblique and transverse sinus to effectively isolate the PV and PW with a single continuous lesion. One advantage of a bilateral approach is that superior vena cava (SVC) can also be addressed in the same procedure [53]. A unilateral thoracoscopic approach can be left- or right-sided. Potential advantages of a left-sided approach include having the tissue dissection away from the heart instead of towards, direct visualization of the left atrial appendage (LAA), and a larger lung capacity during single right-lung ventilation. On the other hand, a right-sided approach permits SVC isolation. In general, a unilateral approach has potential advantages in minimizing postoperative pain, reducing surgical trauma, and avoiding complications in the contralateral side [54]. 

In 41 patients with long-standing PeAF on AADs and without a prior ablation, the HARTCAP AF trial demonstrated that freedom from atrial tachyarrhythmias off AAD was higher in patients undergoing single-staged thoracoscopic hybrid ablation when compared with catheter ablation alone (89% vs. 41%, *p* = 0.002) over a follow-up period of 12 months [55]. The incidence of adverse events was comparable between the two groups (21% vs. 14%, *p* = 0.685). The hybrid ablation lesion consisted of PVI, PWI, LAA exclusion, and, if needed, a cavotricuspid isthmus (CTI) ablation. However, the study had significant differences in baseline characteristics between the two groups, with the catheter ablation arm having a longer AF duration (33 vs. 22 months) [55].

Similar findings were observed in the CEASE AF trial, the largest prospective, multicenter, randomized controlled trial of 154 patients (102 in the hybrid ablation arm and 52 in the catheter ablation arm) with PeAF and long-standing AF [56]. A staged thoracoscopic hybrid ablation achieved similar results for freedom from atrial tachyarrhythmias at 12 months post-procedure (71.6 vs. 39.2, *p* = 0.001). A subgroup analysis further showed an efficacy of 72.7% vs. 41.9% (*p* = 0.002) in PeAF and 66.7% vs. 25% (*p* = 0.09) in long-standing AF. Although adverse safety events (ASE) were numerically higher in hybrid ablation, there was no statistical difference between them (HA = 7.8% (8/102) and CA = 5.8% (3/52), *p* = 0.75). Moreover, PWI along with PVI in patients in the CA arm did not improve efficacy compared to PVI only (40.0% (8/20) versus 38.7% (12/31)). In a 10-year single-center retrospective cohort of 120 patients, the majority being PeAF and long-standing PeAF (20% paroxysmal AF), the atrial-tachyarrhythmia-free survival rates after a single one-step thoracoscopic hybrid ablation procedure at 2 years and 5 years post-procedure were 67.5% and 45.8%, respectively [57].

#### 2.3.2. Hybrid Convergent Ablation 

The subxiphoid approach is the latest of the strategies—also known as a hybrid-convergent procedure—that is less invasive and can be performed in a single stage. In this procedure, the pericardium is accessed directly by a unipolar RF catheter with suction via a transdiaphragmatic/subxiphoid approach. This is a promising technique with an easier learning curve and avoids potential thoracic complications. This technique also allows for LAA ligation under direct visualization [58]. While a thoracoscopic approach might offer some advantages in treating PeAF, the convergent approach using subxiphoid access minimizes surgical trauma to the thorax. One possible disadvantage of this procedure is that it does not allow for complete Ligament of Marshall (LOM) isolation, which can be easily achieved using the thoracoscopic approach [53]. The transdiaphragmatic approach has been associated with increased rates of periprocedural complications, leading to subxiphoid access becoming the preferred modality [59]. Several changes in the technique have occurred over the years due to technological advancements, such as the development of the fourth-generation Epi-Sense device equipped with a sensing function that can differentiate types of tissue on contact. 

The CONVERGE trial showed that a convergent procedure using subxiphoid/transdiaphragmatic access to the pericardium achieved > 90% reduction in the AF burden compared to catheter-only ablation (74% vs. 55%) at 18 months [60]. Hybrid-convergent ablation had superior effectiveness compared to endocardial catheter ablation in PeAF and long-standing AF, with freedom from atrial arrhythmia on AADs at 67.7% versus 50.0%, respectively (risk ratio, 1.35; *p* = 0.036), and an off-anti-arrhythmic-drugs success of 53.5% versus 32.0%, respectively (risk ratio, 1.67; *p* = 0.0128). A potential limitation of this study is that while the hybrid ablation arm patients received epicardial PW silencing, the CA arm did not receive comprehensive PWI endocardially [60]. A meta-analysis estimating the effectiveness of hybrid convergent ablation found a freedom-from-arrhythmia (FFA) rate of 69% (95% CI = 61%–78%, *n* = 523) irrespective of AAD use, and 50% (95% CI = 42%–58%, *n* = 343) off AADs at 1 year or later [61]. Furthermore, after convergent ablation at 18, 24, and 36 months, the FFA rates were 74%, 74%, and 61%, respectively, in patients with PeAF who underwent PVI + PWI followed by an endocardial ablation [62].

A pooled meta-analysis of 16 studies (*n* = 1242) found a mid-term (mean follow-up of 31.5 months) FFA of 74.6% and 65.4% off AAD in patients who underwent hybrid ablation. The FFAs at 1, 2, and 3 years were 78.2%, 74.2%, and 73.6%, respectively. A subgroup analysis further revealed that the thoracoscopic approach is more effective in the treatment of AF, with an FFA of 79.5% as opposed to 54.5% for the hybrid-convergent approach, with no significant differences in complication rates [52]. However, the CASA-AF trial demonstrated that thoracoscopic surgical ablation was not superior to catheter ablation for the treatment of long-standing PeAF in improving arrhythmia-free survival at 12 months post-procedure [63]. Furthermore, thoracoscopic ablation led to a lower degree of symptom improvement, quality-adjusted life years gained at 12 months, and was significantly more expensive than catheter ablation. These results were reiterated in the three-year follow-up of participants of the CASA-AF trial [64]. 

## 3. Non-Pulmonary Vein Triggers (NPVTs)

### 3.1. Left Atrial Posterior Wall

Current guidelines recommend PVI as the standard ablation procedure for rhythm control in symptomatic AF after failure or intolerance to AADs. However, the recurrence rate of arrhythmia remains significant (40–50%) in patients with PeAF [65]. The limited success of PVI in PeAF necessitated the development of novel ablation techniques targeting non-PV targets and atrial substrate modifications, including adjunctive PWI. 

**Figure 2:**
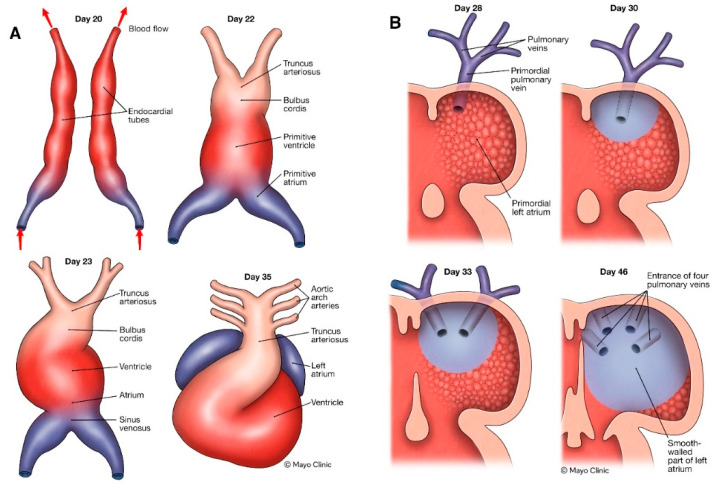
A chronological illustration of (**A**) the embryological development of the heart and great vessels and (**B**) the embryological development of the left-atrial posterior wall and pulmonary veins.

Although the STAR AF II trial results failed to show any additional benefits from atrial substrate modification in patients with PeAF, PWI was not included as an ablation strategy [2]. The PW of the left atrium houses the septopulmonary bundle and shares embryological origin with primordial PVs, hence its arrhythmogenic potential [66,67,68]. The anatomical structure of PV antra and PW exhibit significant heterogeneity in myocardial fiber orientation, which likely results in local reentry circuits due to anisotropic conduction. During development, the myocardium forming the local walls of the left-atrial antrum and dorsal mesocardium (mediastinal myocardium) is different from the myocardium forming walls around the systemic venous structure (primary myocardium) [69] (Figure 2). The mediastinal myocardium is a specialized rapid-conducting-type tissue that is positive for junctional protein connexin-40 and negative for natriuretic precursor peptide. Electrophysiologically, these myocytes are characteristically known to have stronger calcium transients, lowered resting membrane potential, reduced inward rectifier potassium, and larger late sodium currents which result in shorter action potential durations and slower phase 0 upstroke velocities [70]. The current literature proposes that the septopulmonary bundle can contribute to the maintenance of PeAF. The addition of linear lesions along the roof of the left atrium to interrupt the septopulmonary bundle has been used but resulted in a suboptimal clinical response: it was speculated that a durable bidirectional block along the roof is difficult to achieve due to muscle sleeves on the epicardial side [69]. As such, adjunctive PWI evolved, and nonrandomized studies suggested promising outcomes [70,71,72]. According to the current expert consensus statement, PWI could be considered for initial or repeat ablation for PeAF or long-standing PeAF (Class IIb), while ablation of an identified reproducible focal trigger outside PV ostia (Class IIa) is more strongly recommended [73]. 

The CAPLA randomized clinical trial involved 338 patients with PeAF of less than three years duration who already failed one or more AADs. The trial investigated the effectiveness of adjunctive PWI to standard PVI [47]. It showed no significant difference in 1-year freedom from PeAF for groups with PVI and PWI compared to those with PVI alone (52.4% vs. 53.6%, respectively; hazard ratio (HR): 0.99) [47]. A recent meta-analysis of randomized controlled trials showed that adding PWI led to a significant decrease in AF recurrence among patients with PeAF compared to controlled approaches but did not decrease the overall atrial arrhythmia recurrence due to a comparable increase in the risk of atrial flutter [74]. Additionally, although the POBI-AF study (217 patients) did not note significant improvement in patients undergoing circumferential PWI and PW box isolation (POBI) versus CPVI alone (23.8% and 26.5%, respectively; *p* = 0.779), of the 13 patients that had repeat ablation, 85% had a reconnected PVs, and 66% of those in POBI group had a reconnected PW [75]. This shows that complete PWI is extremely technically challenging to perform durably with a radiofrequency catheter. 

Results of the CONVERGE trial (discussed above) further suggest that the LAPW is an attractive target of ablation strategies [60]. Contrastingly, however, a retrospective analysis of the MANIFEST-PF registry of 547 PeAF patients undergoing PFA demonstrated that adjunctive PWI + PVI did not improve freedom from any atrial arrhythmia through a 12-month follow-up as compared with PVI alone in both the full (66.4% vs. 73.1%, respectively; *p* = 0.68) and propensity-matched (71.7% vs. 68.5%, respectively; *p* = 0.51) cohorts [76]. Ongoing trials such as the CORNERSTONE AF, a prospective, multicenter, randomized trial, will provide further insights into the efficacy and safety of adjunctive PWI. 

Rates of acute adverse outcomes and procedural complications are comparable between PVI + PWI and PVI alone, although procedural times may be longer with PVI + PWI [47,74,75]. This was also observed in a pooled meta-analytic analysis of vascular access injuries, pericardial effusion, pericarditis, and stroke/TIA comparing the two groups [44]. Lower rates of procedural complications have also been reported using PFA (PVI + PWI 2.2% vs. PVI only 1.4%) for PWI when compared to thermal ablation techniques [45]. That being said, the proximity of the esophagus to the posterior wall and the need for extensive ablation to achieve exit block (the endpoint of PWI) have raised concerns about the increased risk of atrioesophageal fistula when PWI is performed using thermal ablation energy. 

### 3.2. Left Atrial Appendage (LAA)

Left atrial appendage electrical isolation (LAAEI) has emerged as a potential target of ablation in addition to PVI in PeAF. Di Biase et al. initially demonstrated that the LAA served as the origin of atrial tachyarrhythmias in 27% of patients undergoing repeat procedures for AF [77]. Isolation of the LAA during interventions has been associated with improvement in freedom from AF, particularly in cases where arrhythmias originating from the LAA are induced during the procedure [2]. Findings from the Left Atrial Appendage Isolation in Patients with Long-standing Persistent AF Undergoing Catheter Ablation (BELIEF Trial) compared standard ablation (PVI with supplementary ablation as required) and standard ablation augmented by empirical LAAEI [78]. After 12 months, 56% of patients who received the empirical LAAEI remained free from AF recurrence, compared with 28% of patients who underwent standard ablation alone.

On the other hand, in the context of surgical ablation, Romanov et al. found no decrease in AF recurrence when LAA excision was carried out in conjunction with PVI and box lesion [79]. A permanent LAA excision was pursued, and the results are thought to be more definitive when compared to the BELIEF trial, where the improved results in the LAAEI group could be credited to more extensive ablation and substrate modification [78]. More recently, the aMAZE trial reported no additional benefit of LAA ligation when added to standard PVI in non-paroxysmal atrial fibrillation. In this study, 610 patients were randomized in 2:1 fashion to undergo LAA ligation using the Lariat device in addition to PVI vs. PVI alone. At 12 months follow-up, the absolute rate of freedom from atrial arrhythmia was similar between the 2 groups (63.7% vs. 59.3%, respectively) [80]. 

It is important to consider the risk of complications when pursuing adjunctive LAAEI during catheter ablation. The thin wall of the LAA may be prone to perforation, hence ablation should target the thicker tissue at the LAA ostium. Conducting initial high-output pacing at the distal aspect of the LAA before ablation can serve as a preventive measure against left phrenic nerve injury [81].

Rilling et al. reported that LAAI was associated with a higher incidence of LAA thrombus and stroke, despite oral anticoagulant therapy, compared to a matched control group without LAAI [82]. Romero et al. reported on the enduring effects of LAAEI in individuals with non-paroxysmal AF in a propensity score-matched analysis [81]. Although empirical LAAI decreased AF recurrence without raising acute procedural complications, it was associated with a higher overall rate of thromboembolic events at 5-year follow-up (2.7% vs. 0.7%, *p* = 0.01). This concern has not been validated in a recent metanalysis evaluating the long-term safety and efficacy of LAAI in 2336 patients (LAAEI 3% vs. standard ablation 1.6%; RR 1.76; 95% CI 0.61–5.04; *p* = 0.29) [83]. Despite the conflicting data on thromboembolic risk, implementing post-LAAI stroke prevention strategies, such as lifelong uninterrupted oral anticoagulation, and considering LAA occlusion would be important [81]. It is our practice to evaluate the LAA-emptying function by transesophageal echocardiographic imaging after LAAI for further risk stratification and we have a low threshold for percutaneous occlusion in patients demonstrating persistent contractile dysfunction of LAA after isolation.

Despite the initial enthusiasm, the data on LAAI remain limited to small studies and larger, long-term, and thus randomized, studies are needed to validate the safety and efficacy of this approach. 

### 3.3. Superior Vena Cava (SVC)

The body of research on SVC involvement in AF has demonstrated mixed outcomes regarding its role in arrhythmia propagation. Tsai et al. identified the SVC as a source of premature atrial contractions triggering episodes of paroxysmal AF [84]. Altering autonomic tone via phenylephrine injection effectively suppressed focal AF originating in the PVs but had no such effect on cases originating in the SVC [85]. Goya et al. confirmed detectable potentials within the SVC and breakthrough sites from the RA using electroanatomic mapping [86]. Kholová et al. noted the SVC’s predisposition to automaticity and triggered activity due to the presence of atrial myocytes [87]. 

Meanwhile, evidence on the benefit of SVC isolation (SVCI) in PeAF is conflicting [88]. Meta-analyses by Li et al. and Sharma et al. highlight the debate over its efficacy, with the former reporting improved outcomes and the latter finding no added benefit [89,90]. Yoshida et al. supported the PVI plus SVCI strategy, particularly for PeAF of shorter durations [91]. However, the recent study by Dong et al. indicated that SVCI guided by electroanatomical mapping did not enhance the success rate for paroxysmal AF ablation in the absence of identifiable SVC triggers [92]. 

Therefore, SVCI should be considered case-by-case, particularly for patients with identifiable SVC triggers; for patients with PeAF or those resistant to PVI, incorporating SVCI may offer additional therapeutic value. Utilizing techniques like adenosine injection, isoproterenol infusion, and cardioversion to identify SVC triggers during AF ablation is crucial. Moreover, continuous monitoring of sinus node functionality during these techniques—especially isoproterenol infusion—is essential to avoid collateral injury [88]. These findings highlight the need to better understand the role of SVCI in PeAF and develop personalized approaches based on individual patient characteristics and trigger locations to optimize outcomes.

### 3.4. CFAEs, Rotors, and Dispersion 

The finding that CFAEs represented electrophysiologic substrates of AF led to the hypothesis that their ablation—irrespective of location—may terminate AF and maintain sinus rhythm [93]. Although their definition and detection methods have varied between studies, CFAEs can be identified either by visual inspection or automated depiction of target sites on electroanatomic maps [94]. 

However, randomized trials (e.g., STAR AF II) have mostly failed to show an overt benefit to CFAE ablation as either a stand-alone or as adjuvant to PVI for paroxysmal and PeAF [2]. While an initial meta-analysis of seven RCTs by Li et al. reported an overall benefit of CFAE ablation + PVI in PeAF but not paroxysmal AF, a subsequent meta-analysis of 13 studies (9 RCTs and 4 observational studies) by Providencia et al. demonstrated no benefit to CFAE ablation in both paroxysmal and PeAF, noting that only two of the included studies documented a benefit [95,96]. Given the fundamental questions surrounding CFAEs, such as their definition and their best detection method (with each method displaying poor concordance with other methods [97]), CFAE ablation has not been widely taken up as an adjuvant ablation strategy with PVI. 

Along similar lines, the formation of rotors (i.e., re-entry circuits) in the left atrium has been implicated in the development of PeAF. The focal impulse and rotor modulation (FIRM) methodology is a form of intracardiac panoramic mapping where 64 basket-like electrodes simultaneously contact the atrial endocardium to spatiotemporally map rotors that may be serving as sources of PeAF, thereby rendering them amenable to ablation. Initial observational data such as the CONFIRM study demonstrated that incorporating FIRM mapping-guided ablation in addition to PVI led to significantly lower recurrence of AF in cohorts of paroxysmal and non-paroxysmal AF as compared with PVI alone [98,99,100]. However, randomized trials largely failed to reproduce this benefit as a stand-alone or adjunctive procedure in patients with paroxysmal and PeAF [101,102,103,104]. 

Alternatives to the FIRM methodology for identifying rotors have emerged, including other panoramic mapping techniques (e.g., electrocardiographic mapping and non-contact charge density mapping) and local high-density mapping (e.g., dispersion mapping). While a detailed discussion of the different mapping techniques is beyond the scope of this review (see [105]), electrogram dispersion-guided ablation as an adjunct to conventional PVI significantly improved AF termination rates and recurrence-free survival in patients with paroxysmal and persistent AF [106]. 

Atrial electrograms exhibit spatiotemporal dispersion, regardless of whether they are fractionated or not. These dispersion regions have been shown to strongly correspond with AF driver sites. On the other hand, the majority of the surface area of CFAEs can be located in non-dispersion, bystander regions that do not drive PeAF, and about 30% of the surface area of dispersion regions is composed of non-fractionated electrograms; this may explain the conflicting findings surrounding the efficacy of CFAE ablation [107]. 

Seitz and colleagues presented preliminary evidence of the clinical utility of ablation of electrogram dispersion sites in a prospective observational study of 105 patients with paroxysmal, persistent, and long-standing PeAF admitted for ablation. The electrical activities in both atria were visually mapped using a 20-pole PentaRay catheter, and only those areas exhibiting electrogram dispersion were ablated without PVI [107]. This approach led to AF termination in 95% of patients, with an arrhythmia-free survival of 85% over an 18-month follow-up period in the study population; in comparison, recurrence and recurrence-free survival rates in the validation cohort were 41% and 59%, respectively [107]. Another prospective observational study on an independent cohort of patients with paroxysmal, but not persistent, AF patients demonstrated significantly higher AF termination rates and arrhythmia-free survival at 6 months follow-up compared to patients solely receiving PVI [106]. A randomized controlled trial by Hu et al. randomized 124 obese people with PeAF into conventional PVI and driver ablation of areas of electrogram dispersions [108]. The rate of AF termination in the driver group was significantly higher than in the group undergoing conventional ablation (82.9% vs. 22.8%, respectively; *p* < 0.001). During a median follow-up of 16.9 months, the driver ablation group also had significantly better AF-free survival (91.9% vs. 79.0%, respectively; *p* = 0.026) and AF/atrial tachycardia-free survival (83.9% vs. 64.5%, respectively; *p* = 0.011) than the conventional ablation group. 

Detection of dispersion electrograms can also be automated through artificial intelligence. Seitz and colleagues developed a machine learning algorithm named VX1 (Volta Medical, Marseille, France) trained to identify dispersion electrograms for ablation in a cohort of patients with atrial fibrillation, 29% of which were patients with long-standing PeAF [109]. This approach significantly reduced the operator dependence and variability of CFAE ablation outcomes between centers [109]. Experiences with this AI software have been reported in isolated case reports, demonstrating favorable AF termination rates and arrhythmia-free survival rates [110,111]. In 50 patients with long-standing PeAF undergoing PVI + VX1-guided ablation of spatiotemporal dispersions, Bahlke et al. reported a 78% and 88% freedom of AF after a single and 1.46 procedures in patients with long-standing PeAF [112]. Bentancur-Gutierrez et al. also recently reported their real-world experience using the VX1 software (Marseille, France) in a single-center retrospective cohort study of 16 patients with PeAF (5/16 long-standing PeAF). The rate of PeAF termination to sinus rhythm during ablation was 43.3%, with 87.5% of participants not having any AF recurrences at a 3-month follow-up [113]. More recently, the randomized, multicenter TAILORED-AF trial evaluated the efficacy of adjunctive VX1-guided dispersion ablation + PVI versus PVI alone in patients with PeAF and showed encouraging results with greater freedom from AF (88 vs. 70%) compared to PVI alone (NCT04702451). 

Although the application of artificial intelligence and specifically machine learning in predicting the risk of and detecting AF has been explored, its role in guiding AF ablation is in the early stages [114]. Further research is needed to explore the outcomes of artificial-intelligence-guided ablation strategies in a diverse patient population with variable clinical and disease characteristics and using different ablative technologies. 

### 3.5. Vein of Marshall Ethanol Ablation

The embryonic left anterior cardinal vein involutes in utero as the venous system develops, leaving behind the Ligament of Marshall (LOM) as its remnant. The Vein of Marshall (VOM), anatomically positioned between the LAA and left PVs, is a component of the LOM that courses over the epicardium of the posterior aspect of the LA and drains blood from the LA into the great cardiac vein at the point where it becomes the coronary sinus [115,116]. The VOM contains numerous myocardial tracts—called the Marshall Bundles—that insert into the LA-free wall and coronary sinus [117]. Although the PVs are the most common triggers of AF [118], the Marshall bundles contain rich autonomic innervations and are electrically active, acting as an arrhythmogenic substrate that can trigger/sustain AF [119,120]. This was first shown in canine studies where high-frequency stimulation of the LOM generated both focal and re-entrant electrical circuits that induced AF [120,121,122]. Ectopic beats originating from the VOM area have also been demonstrated in humans with AF [123,124]. 

The VOM can be ablated by retrograde cannulation of the VOM through the coronary sinus and subsequent ethanol infusion. The feasibility of this approach was first demonstrated in canine models and then in 5 human cases [125]. The same group then translated these findings into the Vein of Marshall Ethanol for Untreated Persistent AF (VENUS) trial [126]. This multicenter randomized controlled trial enrolled 343 patients with PeAF to receive additional VOM ethanol ablation (*n* = 185) or catheter ablation alone (*n* = 158). VOM ethanol ablation was successfully performed in 84% of patients. The primary outcome of freedom from AF or atrial tachycardia at 6 and 12 months after a single procedure was achieved in 49.2% of the treatment combined group versus 38% in the PVI group (difference, 11.2%; 95% CI, 0.8%–21.7%; *p* = 0.04) [126]. Notable adverse events reported were vascular access complications (1.9% in VOM + CA vs. 3.8% in CA alone), intraprocedural pericardial effusion (0.6% vs. 0.6%), subacute pericardial effusion/pericarditis not requiring drainage (6.5% vs. 3.8%), and stroke/TIA (1.3% vs. 2.5%). None of the six deaths in the study were related to the VOM procedures [126]. 

VOM ablation has also a significant role during repeat ablation especially in patients who present with peri-mitral atypical atrial flutter. The VOM runs on the epicardial aspect of the posterior mitral isthmus, which is the source of peri-mitral flutter that can account for up to ~50% of AF recurrence post-PVI [127]. The Marshall bundles are also consistently involved in focal or re-entrant circuits formed during peri-mitral flutter [128,129]. Complete mitral isthmus ablation is difficult to achieve with catheter ablation because of its thick myocardium and requires additional epicardial ablation from the coronary sinus. Even with this combined approach, the durability of the mitral isthmus block is suboptimal. Hence, cannulating the VOM and retrogradely injecting ethanol from the epicardium to the endocardium could provide complete ablation of arrhythmogenic myocardial fibers to achieve higher acute and long-term mitral isthmus block [130,131]. Factors associated with higher success rates of mitral isthmus block include male gender, age >60 years, left atrial diameter <55 mm, and AF duration <3 years [132]. 

Derval and colleagues developed a three-step ablation approach strategy, termed the Marshall-PLAN, for the treatment of PeAF consisting of PVI, followed by ethanol infusion to the VOM, and then linear ablation to achieve block across the mitral isthmus, dome, and CTI [133]. This strategy was first employed in a case series of 10 patients who completed the lesion set and were free from arrhythmias through a six-month follow-up [133]. A prospective 12-month follow-up of 75 patients with a primary endpoint of freedom from AF/atrial tachycardia at 12 months who received the Marshall-PLAN lesion set showed that 72% (54 patients) were arrhythmia-free. Of the 68 patients who completed the Marshall-PLAN lesion set, the success rate after a single procedure was 79%, and in patients who underwent 1–2 procedures, it was 89% [134]. Building on these findings, a randomized, controlled, parallel-group, superiority trial of 120 patients with PeAF compared arrhythmia-free survival at 12 months with the Marshall-PLAN strategy versus PVI, demonstrating initial favorable results with arrhythmia recurrence in 8 vs. 18 patients, respectively (*p* = 0.026), translating to success rates of 87% versus 70%.

### 3.6. Low-Voltage Areas

Numerous studies, mostly small and non-randomized, have suggested a role for low-voltage myocardium ablation in enhancing PeAF ablation efficacy. Low-voltage areas (LVAs) are identified through electroanatomical mapping (EAM), and while most studies have used a threshold of <0.5 mV, thresholds of <0.1 mV and <1.0 mV have also been proposed, with higher cut-offs likely implicating a larger LA area and more extensive ablation. A multicenter, randomized, prospective trial (*n* = 450) comparing the outcomes of different ablation strategies for PeAF (PVI with subsequent anatomical guided ablation OR electrogram guided ablation OR extensive electro-anatomical guided ablation) found that electro-anatomical guided ablation was associated with highest freedom from AF recurrence (*p* = 0.002) and highest rate of AF termination, with no significant difference in safety endpoints between groups (*p* = 0.924) [135].

The VOLCANO RCT did not find an AF-recurrence-free survival benefit with LVA ablation in paroxysmal AF patients post-PVI [136]. An extended follow-up analysis of the VOLCANO trial at 25 months noted that AF/AT recurrence rates were comparable between patients with LVAs who underwent LVA ablation and those who did not [137]. The STABLE-SR-III RCT (*n* = 438) compared circumferential PVI (CPVI) + LVA ablation with CPVI alone in older patients with paroxysmal AF and noted a lower AT recurrence rate with CPVI + LVA ablation (31/209 patients, 15%) compared with CPVI alone (15% vs. 24%; HR 0.61; *p*  =  0.03) [138]. Adverse events in the CPVI + LVA group included vascular access complication (1/209) and one death at 25 months due to stroke [128]. 

The recent ERASE-AF trial was pivotal in offering an individualized approach to the ablation of non-paroxysmal atrial fibrillation by targeting areas of low voltage, with significant improvement in clinical outcomes. This trial randomized 324 patients with PeAF to PVI  +  LVA ablation or PVI alone and demonstrated the superiority of adjunctive LVA ablation with a lower rate of AT recurrence (35% vs. 50%; HR 0.62; *p*  =  0.006) [139]. There was a trend toward increased risk of complications in the PVI + LVA group, comprising 10 vascular access events and 2 episodes of cardiac tamponade. Of note, only around a third of patients in the intervention group actually had LVAs and underwent PVI + LVA ablation; hence, two-thirds underwent PVI-alone. Preliminary results from SOLVE-AF, a prospective randomized trial, suggest a significantly higher AF/AT-free survival in PeAF patients undergoing CPVI + LVA ablation compared to CPVI alone (*p* = 0.02), with final results expected in the second half of 2024 [140]. 

Low-voltage-guided ablation of the PW in PeAF has also been studied. A retrospective review of 152 patients who underwent either standard ablation (PVI alone or PVI  +  PW ablation based on physician discretion) or voltage-guided ablation (PVI and addition of PW ablation only if PW low voltage was present) noted a significantly higher freedom from AT recurrence at a 5-year follow-up with voltage-guided ablation (64% vs. 34%; HR 0.358; *p*  <  0.005) [141].

Pooling existing data, a 2022 systematic review by Moustafa et al. (11 studies, *n* = 1597) including non-paroxysmal AF patients found a significantly lower AF recurrence at 1 year with LVA ablation vs. non-LVA ablation (RR 0.63 (27% vs. 36%), 95% confidence interval [CI] 0.48–0.62, *p*  <  0.001], with comparable adverse event rates (RR 0.7 [4.3% vs. 5.4%], 95% CI 0.36–1.35, *p*  =  0.29) [142].

There is promising evidence that the ablation of LVAs, in conjunction with PVI, may improve ablation efficacy and AT-free survival, although large, randomized studies with long-term follow-up are needed to consolidate the clinical evidence to support this approach. 

### 3.7. Crista Terminalis 

Several studies have demonstrated the role of crista terminalis (CT) in both the onset and continuation of AF [143,144,145]. Notably, patients with atrial tachyarrhythmia originating from the CT are more likely to have an older age at presentation, to be female, to have coexistent AVNRT, and have their AT be more likely to be induced by programmed stimulation [146]; as patients with CT-induced focal atrial tachycardia can go on to develop AF, the CT may be functioning as an NPVT [145]. Interestingly, a recent study reported a high prevalence rate of NPVTs in the CT (47.3%) in patients with cardiac amyloidosis who developed AF [147]. 

From a therapeutic standpoint, however, a trial of 66 patients with long-standing PeAF who failed RFA of CFAEs in the left atrium demonstrated no incremental benefit of RFA of right atrial CFAEs, most of which (69%) were located in the CT [148]. In the RASTA randomized controlled trial, Dixit et al. proposed that including adjuvant substrate modifications, including targeting the CT, in addition to PVI, does not enhance the success rate of a single procedure in patients with PeAF [149]. Therefore, although the retrospective cohort study by Morris et al. demonstrated high atrial tachyarrhythmia termination rates (92.2%) and recurrence-free survival over long-term (~7 years) follow-up [146], randomized trials have largely failed to demonstrate a benefit for ablation of driver sites in the CT in the setting of PeAF. Serious adverse event rates were comparable between the CT (4%) and non-CT arms (2% and 8%) in the RASTA trial [149].

## 4. Conclusions

Despite the considerable success achieved in the ablative management of paroxysmal AF using PVI, the optimal ablation strategy in PeAF remains elusive. It is likely that persistent and long-standing persistent atrial fibrillation are common phenotypic manifestations of atrial cardiomyopathy with a heterogenous pathophysiologic basis. In addition, the stage of the disease might not be appropriately captured by the current nomenclature that is dependent only on the duration of atrial fibrillation. Improved success of ablation procedures has been demonstrated clearly in recent studies when AF is targeted early during the disease process [150]. This is critical to consider in our approach to managing these patients and would be the most effective step in improving outcomes in rhythm control, particularly in patients with reduced ejection fraction. Involvement of the cardiac electrophysiologist in the upstream management of many AF patients, in particular those with reduced ejection fraction. 

Despite the signal of benefit noted from some ablation targets in addition to isolating the pulmonary veins, robust evidence is still lacking. It is also important to note that studies investigating additional ablation targets have occurred over different timeperiods, during which we have witnessed considerable advances in ablative technologies, such as the development of contact force-sensing catheters. Our approach continues to rely on PVI during the first procedure along with assessment of LVAs using sinus rhythm mapping to determine the level of electrical remodeling. Then, patients are followed closely with a focus on risk factor modifications and consideration for a second procedure after 6 months if continued AF recurrence is encountered. Repeat procedures are focused on ensuring the durability of PVI and targeting any induced arrhythmias as well as areas of low voltage. 

The promise of newer ablation technologies such as PFA remains to be fully realized in the context of the controversies surrounding targeting NPVTs and substrate modification in PeAF ablation and improving freedom from recurrent AF/AT. 

The hope remains that safer ablative technologies with lower complications will pave the way for higher ablation efficacy and durability. PFA demonstrates a superior safety profile, and with further ongoing investigations, such as the FARADISE, admIRE, and ADVENT trials, it may become the primary choice of ablation technology. With the growing applications of machine learning, cardiac electrophysiology is set to benefit greatly from these advances and a tailored approach to ablating non-paroxysmal atrial fibrillation might be within reach. It is with a lot of enthusiasm that we await further clinical data in this realm including spatiotemporal dispersion mapping. 

The ablative management of PeAF epitomizes the complexity of cardiac electrophysiology—garnering a sound understanding of the anatomical basis of arrhythmogenesis, exploring electrocardiographic properties, and utilizing electroanatomical mapping techniques to identify and ablate culprit activity—all done in a way that maximizes efficacy and minimizes patient harm.

## Figures and Tables

**Table 1:** Notable clinical trials on NPVTs and additional substrate modification in AF ablation.

Ablation Target	Study	Study Population	Study Design	Outcome
Left atrial posterior wall (LAPW)	Kistler 2023 (CAPLA trial)	338 symptomatic patients with PeAF	Multicenter, randomized 1:1 to either PVI with PWI (*n* = 170) or PVI alone (*n* = 168)	Addition of PWI to PVI did not significantly improve freedom from AA compared with PVI alone [HR, 0.99 [95% CI, 0.73–1.36]; *p* = 0.98).
	Kim 2015 (prospective RCT)	120 PeAF patients followed for 12 months after RFCA	Linear ablation + CPVI + PWI (*n* = 60) vs. linear ablation + CPVI (*n* = 60)	PWI (+) group had higher AF termination during procedure (*p* = 0.035), and lower AF recurrence on follow-up (17% vs. 37%, *p* = 0.02)
	Tamborero 2008 (prospective RCT)	120 patients with AF	Prospective, randomized to CPVI + PWI (*n* = 60) vs. CPVI alone (*n* = 60)	PWI did not improve freedom from AA recurrence in patients undergoing CPVI. (log-rank test *p* = 0.943)
	Wong 2023 (PEF-HOT trial)	67 patients with PeAF using high-power short- duration ablation (HPSD)	Multicenter, open-label, single-blind, randomized to PVI + PWI (*n* = 39) vs. PVI alone (*n* = 28)	AA recurrence rates did not significantly differ between PVI + PWI and PVI-only groups (25.6% vs. 28.6%; *p* = 0.7895)
Left atrial appendage (LAA)	Di Biase 2016 (BELIEF Trial)	173 patients with long-standing PeAF	Open-label, randomized to empirical LAAEI + extensive ablation (*n* = 85) vs. extensive ablation alone (*n* = 88)	LAAEI improved long-term freedom from AAs without increasing complications. (unadjusted HR: 2.24; 95% CI: 1.3 to 3.8; log-rank *p* = 0.003)
	Lakkireddy 2024 (aMAZE RCT)	404 patients with non-paroxysmal AF	Multicenter, prospective, open-label, randomized 2:1 to PVI + LAA ligation (*n* = 404) vs. PVI alone (*n* = 206)	Freedom from AAs was 64.3% with LAAI + PVI and was not superior to 59.9% with PVI only (difference, 4.3% [Bayesian 95% credible interval, −4.2% to 13.2%]; posterior superiority probability, 0.835)
Superior vena cava (SVC)	Dong 2024 (RCT)	130 patients undergoing paroxysmal AF ablation	Prospective, multi-center, randomized 1:1 to CPVI + SVCI (*n* = 50) vs. CPVI alone (*n* = 50)	In patients without provoked SVC triggers, SVCI, in addition to CPVI, did not increase freedom from atrial tachyarrhythmias (87.9 vs. 79.6%, log-rank *p* = 0.28)
Crista terminalis (CT)	Dixit 2012 (RASTA study)	156 patients with PeAF. Standard approach: PVI + ablation of non-PV triggers identified using a stimulation protocol	Single-center, randomized to either arm 1 (PVI + standard approach), arm 2, (standard approach + empirical ablation at common NPVTs (including CT)), or arm 3 (standard approach + ablation of LA CFAEs)	Freedom from AAs after single ablation was lowest with arm 3 (29%) vs. arm 1 (49%; *p* = 0.04) and arm 2 (58%; *p* = 0.004)
Vein of Marshall (VOM)	Valderrábano 2020 (VENUS Randomized Clinical Trial)	343 patients with PeAF	Single-blinded trial, randomized 1:1.15 to CA alone (*n* = 158) or CA + VOM ethanol infusion (*n* = 185)	Higher freedom from AF/atrial tachycardia with catheter ablation + VOM ethanol ablation vs. catheter ablation alone at 6 and 12 months (difference, 11.2% [95% CI, 0.8%–21.7%]; *p* = 0.04)
Low voltage areas (LVAs)	Kaiser 2024 (RCT)	100 patients with PeAF	Randomized 1:1 to A or B. A: PVI + substrate modification if LVAs present, B: PVI + additional ablation if AF persisted	Freedom from AAs in 34 (68%) patients of group A vs.28 (56%) patients in group B (*p* = ns). Low complication rate in both groups.
	Huo 2022 (RCT)	324 patients with PeAF	Multicenter trial, randomized 1:1 to PVI alone (*n* = 163) vs. PVI + substrate modification (*n* = 161)	Lower first AA recurrence with PVI + substrate modification (35%) vs. PVI-only (50%) (Kaplan–Meier event rate estimates: HR = 0.62, 95% CI = 0.43 to 0.88, log-rank *p* = 0.006)

AF: atrial fibrillation, PeAF: persistent AF, PWI: posterior wall isolation, AA: atrial arrhythmia, PVI: pulmonary vein isolation, RCT: randomized controlled trial, SVCI: super vena cava isolation, CFAEs: complex fractionated atrial electrograms, LAAEI: left atrial appendage electrical isolation, CA: catheter ablation.

**Table 2:** Notable systematic reviews on NPVTs and additional substrate modification in AF ablation.

Ablation Target	Study	Study Population	Outcome
Left atrial posterior wall (LAPW)	Kanitsoraphan et al., 2022	8 RCTs with 1024 patients with AF	Adjunctive PWI decreased AF recurrence compared to controlled approaches (RR 0.88, 95% CI: 0.81–0.96, *I*^2^ = 48.2%, *p*-value 0.004) without decrease in overall AA recurrence (RR 0.96, 95% CI: 0.88–1.05, *I*^2^ = 31.6%, *p*-value 0.393)
Left atrial appendage (LAA)	Romero et al., 2018	7 studies that enrolled a total of 930 patients [mean age 63 ± 5 years; male: 69%].	LAA ablation + standard ablation appears to improve freedom from AAs in patients with PeAF and long-standing PeAF without raising complication risks. [56% relative reduction and 31.6% absolute reduction; RR 0.44, 95% CI 0.31–0.64; *p* < 0.0001]
Superior vena cava (SVC)	Sharma et al., 2017	3 RCTs with 526 participants with AF	No difference in AF recurrence rate between PVI + SVCI versus PVI alone (39% vs. 60%; OR 0.68; 95% CI 0.43–1.07; *p* = 0.73; *I*^2^ = 0%)
Low voltage areas (LVAs)	Rivera et al., 2024	10 RCTs with 1780 patients with AF	Adjunctive LVA ablation significantly reduced recurrence of AA, as compared with conventional ablation (RR 0.76; 95% CI 0.67−0.88; *p* < 0.01)
	Toumpourleka et al., 2023	5 RCTs with 989 participants with PeAF	Higher probability for freedom from arrhythmia with additional LVA substrate modification when compared to conventional AF ablation (OR 1.73; 95% CI 1.03–2.90; *I*^2^ = 0%; *p* = 0.04).

AF: atrial fibrillation, PeAF: persistent AF, PWI: posterior wall isolation, AA: atrial arrhythmia, PVI: pulmonary vein isolation, RCT: SVCI: super vena cava isolation.

## Data Availability

Now new data were created or analyzed in this study. Data sharing is not applicable to this article.
